# Kidney Function Decline and Apparent Treatment-Resistant Hypertension in the Elderly

**DOI:** 10.1371/journal.pone.0146056

**Published:** 2016-01-25

**Authors:** Jean Kaboré, Marie Metzger, Catherine Helmer, Claudine Berr, Christophe Tzourio, Ziad A. Massy, Bénédicte Stengel

**Affiliations:** 1 Inserm U1018, CESP, Villejuif, France; 2 University Paris-Sud, University Paris-Saclay, Villejuif, France; 3 IRSS/Centre Muraz, Bobo-Dioulasso, Burkina Faso; 4 Inserm U897-Epidemiology-Biostatistic, Bordeaux, France; 5 University of Bordeaux, Bordeaux, France; 6 Clinical Investigation Center – Clinical Epidemiology, Bordeaux, France; 7 Inserm U1061, Montpellier, France, University Montpellier I, Montpellier, France; 8 Division of Nephrology, Ambroise Paré University Hospital, Boulogne Billancourt, France; 9 University Paris-Ouest-UVSQ, Paris, France; Mario Negri Institute for Pharmacological Research and Azienda Ospedaliera Ospedali Riuniti di Bergamo, ITALY

## Abstract

**Background:**

Cross-sectional studies show a strong association between chronic kidney disease and apparent treatment-resistant hypertension, but the longitudinal association of the rate of kidney function decline with the risk of resistant hypertension is unknown.

**Methods:**

The population-based Three-City included 8,695 participants older than 65 years, 4265 of them treated for hypertension. We estimated the odds ratios (OR) of new-onset apparent treatment-resistant hypertension, defined as blood pressure ≥ 140/90 mmHg despite use of 3 antihypertensive drug classes or ≥ 4 classes regardless of blood pressure, associated with the mean estimated glomerular filtration rate (eGFR) level and its rate of decline over 4 years, compared with both controlled hypertension and uncontrolled nonresistant hypertension with ≤ 2 drugs. GFR was estimated with three different equations.

**Results:**

Baseline prevalence of apparent treatment-resistant hypertension and of controlled and uncontrolled nonresistant hypertension, were 6.5%, 62.3% and 31.2%, respectively. During follow-up, 162 participants developed apparent treatment-resistant hypertension. Mean eGFR decline with the MDRD equation was 1.5±2.9 mL/min/1.73 m² per year: 27.7% of the participants had an eGFR ≥3 and 10.1% ≥ 5 mL/min/1.73 m² per year. After adjusting for age, sex, obesity, diabetes, and cardiovascular history, the ORs for new-onset apparent treatment-resistant hypertension associated with a mean eGFR level, per 15 mL/min/1.73m² drop, were 1.23 [95% confidence interval 0.91–1.64] compared to controlled hypertension and 1.10 [0.83–1.45] compared to uncontrolled nonresistant hypertension; ORs associated with a decline rate ≥ 3 mL/min/1.73m² per year were 1.89 [1.09–3.29] and 1.99 [1.19–3.35], respectively. Similar results were obtained when we estimated GFR with the CKDEPI and the BIS1 equations. ORs tended to be higher for an eGFR decline rate ≥ 5 mL/min/1.73m² per year.

**Conclusion:**

The speed of kidney function decline is associated more strongly than kidney function itself with the risk of apparent treatment-resistant hypertension in the elderly.

## Introduction

Despite use of an increasing number of antihypertensive medications, uncontrolled hypertension remains common and one of the most important risk factors for end-stage renal disease and cardiovascular mortality in persons with chronic kidney disease (CKD)[1–7]. Uncontrolled hypertension most often results from an inadequate treatment regimen or from non-adherence, but a substantial proportion of patients at particularly high risk develop resistant hypertension [8,9]. True resistant hypertension, defined as uncontrolled blood pressure (BP) ≥ 140/90 mmHg while using ≥ 3 antihypertensive drug classes at optimal doses including a diuretic, or using ≥ 4 classes regardless of BP, is difficult to assess in population-based studies because information about doses and adherence is often lacking [2,10,11]. Prevalence rates of apparent treatment-resistant hypertension (aTRH) of 0.5 to 15% have been observed among people treated for hypertension across the world; these estimates are about twice as high in those with CKD [2,12–16].

Most studies reporting associations between CKD and aTRH have been cross-sectional [2,12,15,17,18] with one exception [19]. They have consistently showed a higher prevalence of aTRH associated with either lower estimated glomerular filtration rates (eGFR) or higher albumin-to-creatinine ratios (ACRs) or both, independent of other major determinants including age, gender, race, smoking, obesity, diabetes, and cardiovascular disease (CVD)[2,12,14,15,17,18]. The longitudinal association between the rate of kidney function decline and the risk of resistant hypertension, however, is unknown. Moreover, although the risk of poor BP control increases greatly with age, few studies have focused on elderly populations [7,14].

We therefore studied the association of kidney function and its rate of decline with both the prevalence of and new-onset aTRH in the elderly population of the Three-City study.

## Materials and Methods

### Study design and participants

**Fig 1:**
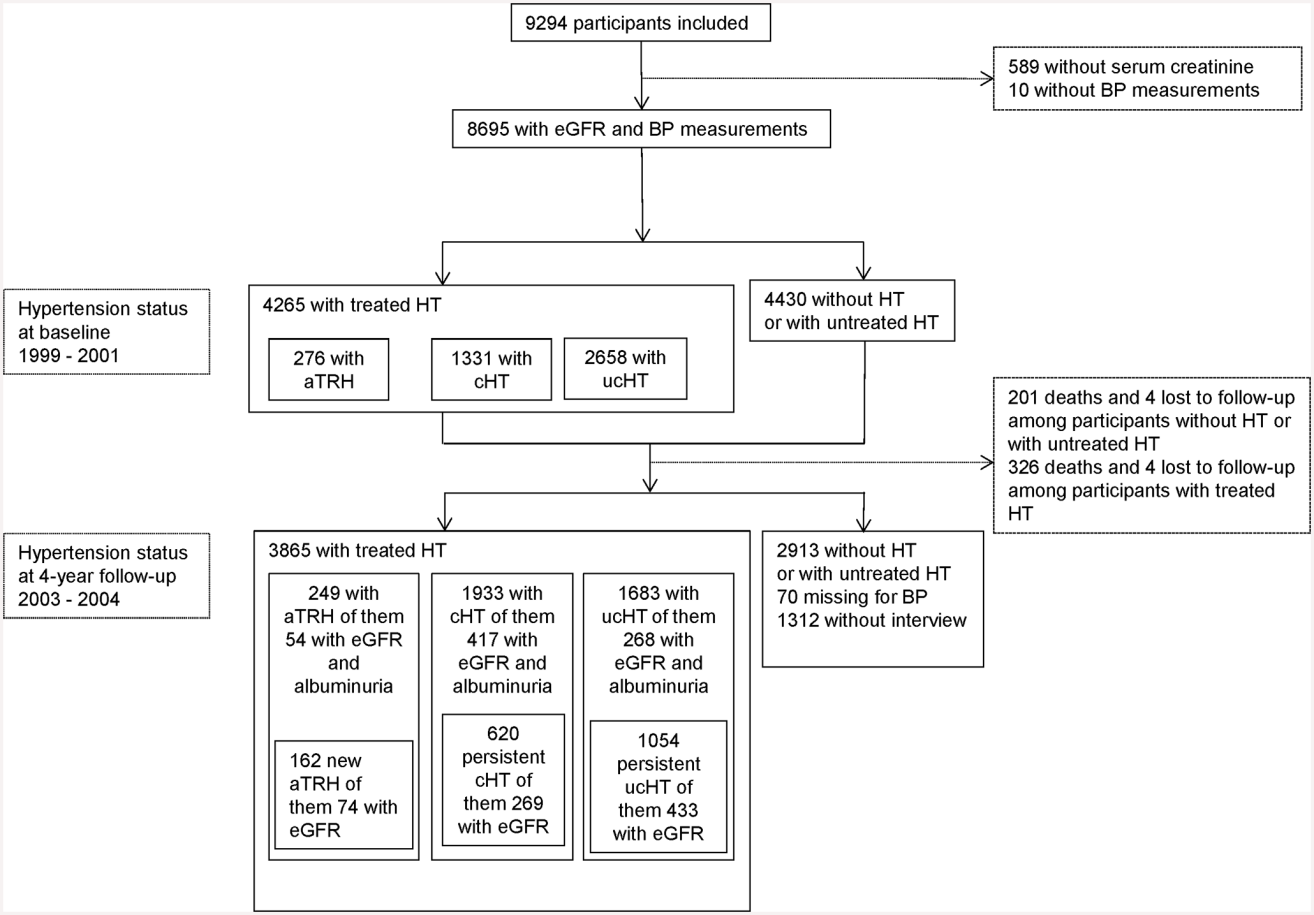
Flowchart of the study participants. **Abbreviations**: eGFR: estimated glomerular filtration rate; BP: blood pressure; HT: hypertension; aTRH: apparent treatment-resistant hypertension; cHT: controlled HT; ucHT: uncontrolled hypertension with ≤ 2 antihypertensive drugs; Persistent cHT: controlled hypertension during the 4-year follow-up; Persistent ucHT: uncontrolled hypertension with ≤ 2 antihypertensive drugs during the 4-year follow-up.

The Three-City study is a population-based prospective cohort that included 9,294 non-institutionalized individuals aged 65 years or older randomly selected from electoral rolls of three French cities from March 1999 through March 2001—Bordeaux (2,104), Dijon (4,931), and Montpellier (2,259). Details of the study protocol have been published elsewhere [20]. Both BP and kidney function were measured at baseline for 8,695 participants, 4,265 of whom had treated hypertension. Of the 8,695 participants with both measurements, 6,848 were examined at the 4-year follow, including 3,865 of those with treated hypertension (Fig 1). The institutional review committee of Kremlin-Bicêtre University Hospital approved the study protocol, and all participants provided written informed consent.

### Data collection

Trained staff administered standardized questionnaires and performed clinical examination at baseline and at the 4-year follow-up. Socio-demographic data, education level, monthly income, smoking status, and medical history were recorded. History of CVD included coronary heart disease, myocardial infarction, heart failure, stroke, peripheral artery disease, and artery surgery or angioplasty. Obesity was defined as a body mass index (BMI) ≥ 30 kg/m². Medication use was collected and coded according to the World Health Organization’s Anatomical Therapeutic Chemical classification system. All three centers collected blood at baseline. For financial reasons and because of the specific objectives in each center, only two centers (Bordeaux and Dijon) collected blood at 4 years and only one (Bordeaux) urine. Hypercholesterolemia was defined as use of statins or fasting cholesterolemia ≥ 6.2 mmol/L. Diabetes was defined as the current use of antidiabetic drugs or fasting glycemia ≥7.2 mmol/L or non-fasting glycemia ≥ 11 mmol/L.

### Assessment of chronic kidney disease and rate of kidney function decline

At baseline, creatinine was measured in a single laboratory with the Jaffé assay and further standardized to isotope dilution mass spectrometry (IDMS) traceable enzymatic creatinine as described elsewhere [21]. We used equations from the Modification of Diet in Renal Disease (MDRD-eGFR), the Chronic kidney Disease epidemiology collaboration (CKDEPI-eGFR), and the Berlin Iniative Study (BIS1-eGFR) to calculate eGFR, without correcting for race, which was unavailable [22–24]. CKD was defined as an eGFR< 60 mL/min per 1.73m² [25].

At the 4-year follow-up, 1,629 of 3,865 participants with treated hypertension had a second creatinine measurement, similarly standardized; 739 also had urine measurements taken to assess protein-to-creatinine ratio (PCR) and ACR when proteinuria was <300 mg/L. Albuminuria was defined as ACR ≥ 3 mg/mmol or PCR ≥ 30 mg/mmol when ACR was missing (n = 23). For each participant, we calculated eGFR slope in mL/min/1.73 m^2^ per year as the difference between 4-year and baseline eGFR divided by the absolute follow-up time.

### Blood pressure measurement and definition of hypertension control status

During both examinations, trained staff using a validated digital electronic sphygmomanometer with an appropriately sized cuff on the right arm (OMRON M4; OMRON Corp., Kyoto, Japan) measured BP twice at the participant’s home, after at least five minutes at rest in seated position [26]. The analysis used the mean of these two measurements. Hypertension was defined as controlled (cHT) if the mean systolic and diastolic BP was < 140/90 mm Hg with ≤3 antihypertensive drug classes, and as uncontrolled nonresistant (ucHT), if it was ≥ 140/90 mm Hg with ≤ 2 drugs; aTRH was defined as uncontrolled BP ≥ 140/90 mmHg in patients receiving ≥ 3 antihypertensive drug classes or ≥ 4, regardless of BP level. We also used a cut-off of 150/90 mm Hg as recommended by JNC8 for the elderly to assess its impact on prevalence estimates [27].

### Statistical analysis

We classified participants into 3 groups according to hypertension control status and used cHT and ucHT as references for aTRH. We first compared baseline characteristics between the group with aTRH and the other two, with Student’s t-test or the Wilcoxon or chi-square test, as appropriate. We also compared the distribution of the 3 groups according to the presence or absence of CKD.

Second, we used multinomial regression models to estimate odds ratios (OR) of prevalent aTRH at baseline, compared with the two reference groups, associated with eGFR treated continuously per 15 mL/min/1.73 m^2^ drop, which is equivalent to the GFR drop between two CKD stages, and adjusted for age, gender, smoking, obesity, diabetes, history of cardiovascular disease, and center. In the subsample of 1,629 participants with two eGFR measurements, we then estimated ORs of prevalent aTRH at the 4-years follow-up associated with an eGFR decline rate ≥ 3 mL/min/1.73 m^2^ per year and adjusted for the same covariates, with comorbidities updated at 4 years. This cutoff was chosen because it is about 3 times greater than the annual physiological kidney function decline due to aging. To account for the phenomenon of regression to the mean [28], we adjusted for a mean eGFR per 15 mL/min/1.73 m^2^ drop, computed as the average of the baseline and 4-year eGFR values. As a sensitivity analysis, we also estimated ORs for an eGFR decline rate ≥ 5 mL/min/1.73 m^2^ per year, which defines fast progression according to the Kidney Disease Improving Global Outcomes (KDIGO) 2012 [29]. Finally, the impact of albuminuria on these associations was analyzed in the subsample of 739 participants with ACR values, after checking that ORs for mean eGFR and eGFR slope were similar in those with and without ACR ratio values before adjustment.

Third, we compared participants with new-onset aTRH, including 162 individuals without aTRH at baseline, to the following stable reference groups: 620 individuals with persistent cHT during follow-up and 1,054 with persistent ucHT (Fig 1). To assess the predictive value of the eGFR at a specific time point for the onset of aTRH, we estimated the adjusted OR of new-onset aTRH associated with baseline eGFR per 15 mL/min/1.73 m^2^ drop, compared with both reference groups. To assess the impact of the rate of kidney function decline, we finally estimated the OR of new-onset aTRH associated with an eGFR decline ≥3 (or ≥5) mL/min/1.73m² per year over the period, adjusted for age, gender, center, mean eGFR per 15 mL/min/1.73m² drop, and comorbidities in the subsample of participants with two eGFR measurements (Fig 1).

We tested the interactions between age, sex, diabetes, and eGFR in all models. All statistical analyses were conducted with SAS 9.3 software (SAS Institute Inc, Cary, NC); all probabilities were two-tailed, and a p-value ≤ .05 was defined as statistically significant.

## Results

### Participant characteristics at baseline

The prevalence of aTRH was 6.5%, that of cHT 31.2%, and that of ucHT 62.3%. When we applied the JNC8 BP cutoffs (≥ 150/90 mm Hg) for participants older than 60 years, these percentages were 5.4%, 46.7%, and 47.9%. The overall mean MDRD-eGFR was 74±17.0 mL/min/1.73m², the mean BIS1-eGFR 63.2±12.1, and the median CKD-EPI-eGFR (quartiles) 75.2(63.9–84.6). The overall prevalence of CKD was twice as high with the BIS1 than with the other two equations. Among participants with aTRH, approximately 2% had controlled BP while taking ≥ 4 drugs. Participants with aTRH were significantly older than those in the other two groups and more often obese; they also had diabetes and CVD more often, as well as higher pulse pressure and social factors that were lower, but not significantly so (Table 1).

**Table 1:** Baseline characteristics of participants according to hypertension control status.

Baseline characteristics	All	aTRH	cHT	p-value aTRH vs cHT	ucHT	p-value aTRH vs ucHT
N	4265	276	1331		2658	
Age, years	75.1±5.6*	76.1±6.0*	74.9±5.5*	0.001	75.1±5.6*	0.008
Men	39.5	46.4	32.0	< 0.001	42.5	0.22
*Blood pressure*, *mm Hg*						
Systolic	150±22*	165±19*	127±10*	< 0.001	161±17*	< 0.001
Diastolic	83±12*	86±12	74±8	< 0.001	87±11	0.071
Pulse pressure	67±17	79±18*	53 ±10*	< 0.001	73±15*	< 0.001
Isolated systolic hypertension	42.7	66.3			61.6	0.124
Low income	63.7	67.4	63.9	0.26	63.3	0.17
Education <12years	66.9	67.0	67.5	0.76	66.7	0.96
Ever smokers	38.5	43.8	35.0	< 0.01	39.8	0.19
Body mass index ≥30kg/m²	18.1	28.3	17.2	< 0.001	17.4	< 0.001
Diabetes	13.8	30.4	10.7	< 0.001	13.7	< 0.001
History of CVD	12.9	27.2	14.2	< 0.001	10.8	< 0.001
Hypercholesterolemia	58.1	58.3	59.3	0.77	57.4	0.77
Statin use	35.8	39.9	35.5	0.18	35.6	0.16
*CKD prevalence according to eGFR equation*						
MDRD-eGFR	19.0	34.8	19.0	< 0.001	17.4	< 0.001
CKD-EPI eGFR	18.5	34.4	18.3	< 0.001	17.0	< 0.001
BIS1-eGFR	38.1	50.7	37.6	< 0.001	37.1	< 0.001
*No of antihypertensive drug classes*						
1	61.2		63.3		66.5	
2	30.3		30.1		33.5	
3	7.6	86.2	6.6			
4	0.8	13.0				
5	0.1	0.7				
RASi	45.6	77.5	41.5	< 0.001	44.4	< 0.001
Calcium channel blockers	26.9	61.2	28.3	< 0.001	22.6	< 0.001
At least one diuretic	31.2	74.6	33.4	< 0.001	26.6	< 0.001
Thiazide diuretics	11.9	12.3	13.7	0.548	11.1	0.541
Potassium sparing diuretics	16.8	18.8	19.7	0.747	15.2	0.108
At least 1 loop-acting diuretic	12.2	46.4	12.9	< 0.001	8.4	< 0.001
β-blockers	34.9	60.5	34.1	< 0.001	32.6	< 0.001
α-β-blockers	0.8	1.8	0.9	0.178	0.7	0.068
α-blockers	9.1	40.5	5.9	< 0.001	7.4	< 0.001
At least 1 vasodilator	25.6	23.6	26.6	0.294	25.4	0.493

**Abbreviations**: aTRH: apparent treatment-resistant hypertension; cHT: controlled hypertension; ucHT: uncontrolled hypertension with ≤ 2 antihypertensive drugs; Isolated systolic hypertension defined as SBP≥140 mmHg; RASi: renin angiotensin system inhibitors; CVD: cardiovascular disease; eGFR: estimated glomerular filtration rate. MDRD: Modification of Diet in Renal Disease study; CKDP-EPI: Chronic kidney disease epidemiology collaboration; BIS1: the Berlin Initiative Study equation 1. Diabetes defined as use of antidiabetics or fasting glycemia ≥7.2 mmol/L or non-fasting glycemia ≥ 11mmol/l; Hypercholesterolemia defined as use of statins or fasting cholesterolemia ≥ 6.2 mmol/L; Low income defined as <34440€/years. CKD: chronic kidney disease defined as an eGFR < 60mL/min per 1.73m². Values are percentages.

* mean ± SD.

**Fig 2:**
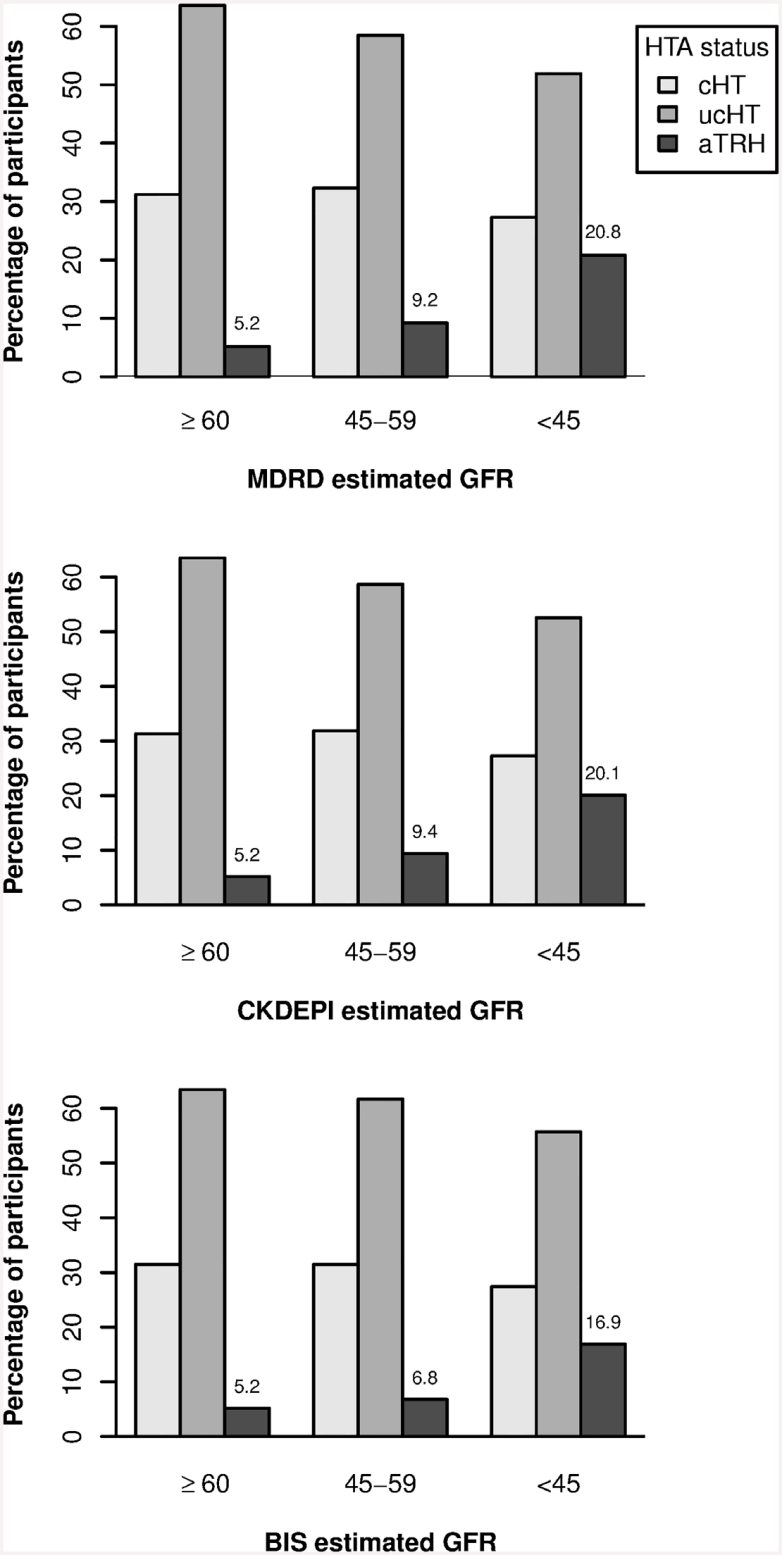
Prevalence of hypertension control status according to GFR estimated from the MDRD, the CKD-EPI and the BIS1 equations at baseline. **Abbreviations**: cHT: controlled hypertension; aTRH: apparent treatment resistant hypertension; ucHT: uncontrolled nonresistant hypertension with ≤ 2 antihypertensive drugs; eGFR: estimated glomerular filtration rate; MDRD: Modification of Diet in Renal Disease study; CKDP-EPI: Chronic kidney disease epidemiology collaboration; BIS1: the Berlin Initiative Study equation 1. eGFR categories in mL/min per 1.73m²: ≥60, 45–59 and <45. p-value for global comparison of frequencies of aTRH, cHT and ucHT according to eGFR levels was < 0.001 for each equation.

They were also more likely to be men and current or past smokers than those with cHT. The prevalence of aTRH increases with the reduction of the level of eGFR and it is 3 to 4 times higher in participants with an eGFR below 45 mL/min per 1.73m² regardless of the equation used (Fig 2).

Around 75% of the participants with aTRH reported taking diuretics and renin angiotensin system inhibitors (RASi), while less than 66% reported calcium channel blockers and betablockers.

### Kidney function and prevalence of apparent treatment-resistant hypertension

At baseline, lower MDRD-eGFR values were associated with higher ORs of aTRH, compared to both reference groups (Table 2, part I), independent of confounders. Using CKDEPI-eGFR or BIS1-eGFR tended to produce stronger ORs (Table A in S1 File).

**Table 2:** Association of kidney function and past decline rate with the prevalence of apparent treatment-resistant hypertension at baseline and at the 4-year follow-up.

	aTRH vs cHT	aTRH vs ucHT
	Adjusted OR [95%CI]	
All participants at baseline: aTRH at baseline	n = 276 vs 1331	n = 276 vs 2658
Age per 5years	1.17 [1.03–1.32]	1.10 [0.98–1.24]
Men	1.72 [1.23–2.41]	0.96 [0.70–1.32]
Ever smokers	0.99 [0.71–1.37]	1.05 [0.77–1.43]
Body mass index ≥ 30 kg/m²	1.80 [1.32–2.46]	1.80 [1.34–2.42]
Diabetes	3.37 [2.44–4.66]	2.61 [1.95–3.49]
History of CVD	1.72 [1.24–2.40]	2.87 [2.10–3.92]
eGFR per 15 mL/min/1.73m² drop	1.29 [1.16–1.48]	1.33 [1.19–1.48]
Participants with eGFR measured at 4 years: aTRH at 4 years	n = 114 vs 823	n = 114 vs 692
Age per 5years	1.00 [0.79–1.28]	0.85 [0.66–1.08]
Men	1.77 [1.07–2.93]	1.05 [0.63–1.74]
Ever smokers	1.44 [0.88–2.35]	1.41 [0.86–2.31]
Body mass index ≥ 30 kg/m²	2.24 [1.40–3.58]	2.20 [1.37–3.53]
Diabetes*	3.77 [2.39–5.96]	3.11 [1.97–4.93]
History of CVD **	0.99 [0.59–1.66]	1.45 [0.85–2.46]
Mean eGFR per 15 mL/min/1.73m² drop	1.23 [0.99–1.51]	1.28 [1.04–1.59]
eGFR decline ≥ 3 mL/min/1.73m²	1.77 [1.17–2.70]	2.06 [1.35–3.16]
Participants with eGFR and albuminuria: aTRH at 4 years	n = 54 vs 417	n = 54 vs 268
Age per 5 years	0.75 [0.53–1.07]	0.68 [0.47–0.97]
Men	1.11 [0.60–2.07]	0.93 [0.49–1.77]
Diabetes	5.32 [2.81–10.0]	4.93 [2.53–9.62]
Mean eGFR per 15 mL/min/1.73m²drop	1.15 [0.87–1.53]	1.19 [0.89–1.59]
eGFR decline ≥ 3 mL/min/1.73m²per year	1.56 [0.85–2.88]	1.91 [1.02–3.60]
Albuminuria	2.49 [1.20–5.18]	1.95 [0.92–4.13]

All analyses were adjusted for center. **Abbreviations**: aTRH: apparent treatment-resistant hypertension; cHT: controlled hypertension; ucHT: uncontrolled hypertension with ≤ 2 antihypertensive drugs; OR: odds ratios; CI: 95% confidence interval; eGFR: glomerular filtration rate estimated using the MDRD equation; MDRD: Modification of Diet in Renal Disease; CVD: Cardiovascular disease. **Definitions**: Diabetes: use of antidiabetics drugs or fasting glycemia ≥ 7.2 mmol/L or non-fasting glycemia ≥ 11 mmol/l;

*Diabetes: updated with reported diabetes status at 4 years; CVD: personal history of cardiovascular diseases;

**CVD: updated with incident non-fatal CVD events between baseline and the 4-year follow-up; Mean eGFR: mean of baseline and 4-year eGFR; eGFR decline calculated as eGFR at 4 years minus eGFR at baseline divided by the absolute number of years.

In the subsample of participants with a second eGFR assessment at four years, the percentage with a decline rate ≥ 3 mL/min per 1.73 m² was 27.7% with the MDRD, 24.3% with the CKDEPI, and 21.1% with the BIS1 equation; these percentages were 10.1%, 8.6%, and 4.4%, respectively for an eGFR decline rate ≥ 5 mL/min per 1.73 m². An MDRD-eGFR decline rate ≥ 3 mL/min per 1.73 m² was associated with a higher OR of aTRH, independent of mean MDRD-eGFR over the period and other covariates (Table 2, part II). We found similar results with the CKDEPI-eGFR and BIS1-eGFR equations (Table B in S1 File). Odds ratios tended to be higher, but confidence intervals wider, with a decline rate threshold of 5 mL/min/1.73m² per year (Table E in S1 File). Similar ORs were found for mean MDRD-eGFR and rapid MDRD-eGFR decline rate in the subsample of those with ACR measurements, which were only slightly reduced by adjustment for albuminuria (Table 2, part III). Albuminuria was associated with a higher OR of aTRH, but this association was statistically significant only when compared with cHT. Using the CKDEPI or BIS1 equations did not change these results (Tables C and F in S1 File).

### Changes in hypertension control status over 4 years

Of the 8,695 participants at baseline, 78% had BP measured at the 4 years follow-up; 15.1% declined participation, BP was missing at examination for 0.8%, and 6% had died (Table 3). At 4 years, 6.4% were classified with aTRH, 50% with cHT, and 43.5% with ucHT. Among those without aTRH at baseline, 162 developed the condition during follow-up; 149 (92%) among aTRH free participants treated for hypertension, i.e., 3.5% over 4 years and 0.5 per 100 person-years (Table 3).

**Table 3:** Changes in hypertension control status over 4-year follow-up.

	Hypertension control status at the 4-year follow-up											
	Known (n = 6778)							Unknown (n = 1847)				
Baseline hypertension control status	noHT	utHT	cHT	ucHT	aTRH	Missing BP n	Sub total n	Alive	Dead	Lost to follow-up	Sub total (n)	Total n
noHT %	70.2	16.2	11.2	2.3	0.1	18	1610	77.2	22.3	0.5	364	1974
utHT %	30.6	38.6	14.6	15.5	0.6	20	1945	76.1	23.5	0.4	511	2456
cHT %	6.6	3.7	62.3	24.7	2.7	7	1003	64.3	35.4	0.3	328	1331
ucHT %	2.0	3.0	38.4	50.7	6.0	22	2098	69.6	30.0	0.4	560	2658
aTRH %	0.0	0.0	29.1	24.9	46.0	3	192	48.8	50.0	1.2	84	276
Total	1815	1098	1933	1683	249	70	6848	1312	527	8	1847	8695

**Abbreviations**: noHT: no hypertension; utHT: untreated hypertension; cHT: controlled hypertension; ucHT: uncontrolled hypertension with ≤ 2 antihypertensive drugs; aTRH: apparent treatment-resistant hypertension. BP: blood pressure.

### Kidney function and new-onset apparent treatment-resistant hypertension

Unlike obesity and diabetes, baseline MDRD-eGFR level was not related to new-onset aTRH, compared to either persistent cHT or ucHT (Table 4, Part I). In contrast, a rapid MDRD-eGFR decline (≥ 3 mL/min/1.73m² per year) was significantly associated with a higher OR of new-onset aTRH, regardless of the reference group and independent of mean MDRD-eGFR over the period and other covariates (Table 4, Part II). Using the CKDEPI or the BIS1 equation did not change these associations (Table D in S1 File). Odds ratios tended to be higher for eGFR decline ≥ 5 mL/min/1.73m² per year (Table G in S1 File).

**Table 4:** Association of kidney function at baseline and kidney function decline rate with new-onset apparent treatment-resistant hypertension.

	aTRH vs persistent cHT	aTRH vs persistent ucHT
	Adjusted OR	
All participants at baseline	n = 162 vs 620	n = 162 vs 1054
Age per 5 years	1.16 [0.97–1.38]	1.02 [0.87–1.21]
Men	2.44 [1.67–3.55]	0.98 [0.69–1.38]
Body mass index ≥30Kg/m²	1.57 [1.02–2.40]	1.69 [1.14–2.52]
Diabetes	3.31 [2.12–5.16]	2.26 [1.53–3.35]
History of CVD	0.75 [0.44–1.28]	1.86 [1.12–3.09]
eGFR per 15 mL/min/1.73m² drop	0.99 [0.83–1.18]	0.98 [0.83–1.15]
Participants with eGFR measured at 4 years	n = 74 vs 269	n = 74 vs 433
Age per 5 years	0.94 [0.69–1.28]	0.90 [0.67–1.21]
Men	2.24 [1.29–3.91]	1.11 [0.66–1.86]
Body mass index ≥30Kg/m²	1.34 [0.71–2.52]	1.65 [0.90–3.01]
Diabetes	3.15 [1.60–6.21]	1.93 [1.06–3.51]
History of CVD	0.84 [0.34–2.11]	1.38 [0.58–3.31]
Mean eGFR per 15 mL/min/1.73m² drop	1.23 [0.91–1.64]	1.10 [0.83–1.45]
eGFR decline ≥3 mL/min/1.73m² per year	1.89 [1.09–3.29]	1.99 [1.19–3.35]

All analyses were adjusted for center. **Abbreviations**: aTRH: incident apparent treatment-resistant hypertension; cHT: controlled hypertension; ucHT: uncontrolled hypertension with ≤ 2 antihypertensive drugs; OR: odds ratios; CI: 95% confidence interval; eGFR: glomerular filtration rate estimated using the MDRD equation; MDRD: Modification of Diet in Renal Disease; CVD: Cardiovascular disease. **Definitions**: Diabetes: use of antidiabetics or fasting glycemia ≥ 7.2 mmol/L or non-fasting glycemia ≥ 11 mmol/l; CVD: personal history of cardiovascular diseases; mean eGFR: mean of baseline and 4-year eGFR; eGFR decline calculated as eGFR at 4 years minus eGFR at baseline divided by the absolute number of years.

## Discussion

In this community-dwelling elderly population, ucHT affected 62% of those treated for hypertension, while only 6.5% had aTRH. The novelty of this study lies in the finding that a rapid decline in kidney function was associated with a higher OR of aTRH independent of eGFR level and other major risk factors for resistant hypertension. Another new element is that kidney function, measured once at baseline, seems to lack predictive value for new-onset aTRH in this population. These findings provide insight about the relation between CKD and resistant hypertension and about the clinical perspectives for managing hypertension in the elderly.

It is difficult to compare our prevalence estimate of aTRH with others, because with few exceptions [18], previous work has studied populations younger than ours [2,12,13,15–17]. Unexpectedly, however, the prevalence, applying a similar definition, was lower than the 12% to 15% found in US [2,12,15] or Spanish adults treated for hypertension [16,18]. One possible explanation is that standardized in-home BP measurements by trained staff reduced white-coat hypertension, which may account for a substantial proportion of aTRH [30]. Nevertheless, this low percentage in our study accounted for less than 10% (9.3%) of all ucHT cases, in contrast with the 25 to 32% observed in most other studies while their percentages of ucHT have been higher [12,31]. Our results are therefore more likely to reflect an underestimation of resistant hypertension due to underprescription than a true low prevalence of resistant hypertension. It may also result from self-selection of healthier elderly participants in this cohort than in the general population. Consistent with this hypothesis, the finding that about 40% of the participants with ucHT at baseline were still uncontrolled on 1 or 2 drugs at 4 years demonstrates substantial treatment inertia (Table 5). In addition, the findings only 46% of the participants with aTRH reported taking loop-acting diuretics sheds light on the underprescription of this therapeutic group, which is nonetheless recommended for resistant hypertension.

**Table 5:** Changes in antihypertensive therapy in 1054 participants with persistent uncontrolled nonresistant hypertension over 4-year follow-up.

Treatment at baseline			Treament at 4 years							
	Number of drugs		CCB		RASi:		Diuretics		Betablockers	
	1 drug	2 drugs	No	yes	no	yes	no	yes	no	yes
Number of drugs % (n)										
1	76.9 (560)	23.1 (168)								
2	17.5 (57)	82.5 (269)								
CCB % (n)										
no			92.0 (750)	8.0 (65)						
yes			27.6 (66)	72.4 (173)						
RASi: % (n)										
no					73.7 (426)	26.3 (152)				
yes					11.1 (53)	88.9 (423)				
Diuretics % (n)										
no							92.2 (718)	7.8 (61)		
yes							25.5 (70)	74.5 (205)		
Beta-blockers % (n)										
no									91.6 (669)	8.4 (61)
yes									13.3 (43)	86.7 (281)

**Abbreviations**: CCB: Calcium channel blocker; RASi: renin angiotensin system inhibitors.

Risk factors most commonly related to aTRH in previous cross-sectional surveys include age, gender, obesity, diabetes, and CKD. We confirm the strong association with diabetes and obesity [13,15,17] and extend these findings by showing the potential predictive value of these two conditions measured at one time point for new-onset aTRH. In contrast, the relation with age and gender is less clear. Only a few studies have shown significant associations between increasing age and aTRH [13,15]. In the current study, men were at higher risk of aTRH compared with the cHT, but not the ucHT group, and that is likely to reflect poorer BP control in men than women, as previously reported in this population [26]. Consistent with several other studies, we also found a prevalence of aTRH about twice as high in participants with than without CKD [12,15,17,18,31].

Note that all three equations yielded similar findings about the associations of CKD with resistant hypertension. However, while using the MDRD and the CKDEPI equations provided similar estimates of CKD prevalence, the estimates with the BIS1 were twice as high. Two studies have assessed the performance of these equations in the elderly population in France [32,33]. Although both agreed that the BIS equation was more accurate than the other two, they disagreed about the magnitude of bias compared to measured GFR. It is nevertheless beyond the scope of this study to conclude which equation is the best.

The major importance of this study is that it provides additional evidence about the longitudinal association between kidney function decline and aTRH. First, it shows that a fast rate of eGFR decline is strongly associated with new-onset aTRH over a 4-year period, independent of mean eGFR level over that period and other comorbidities. Although it was not possible to adjust for albuminuria, which was lacking at baseline, we were able to show that, in the subgroup of participants with measurements at 4 years, past eGFR decline remained strongly associated with prevalent aTRH after adjusting for albuminuria at 4 years as well as other covariates. This is consistent with the study by Tanner et al [17] showing that both lower eGFR and higher albuminuria independently contribute to higher aTRH prevalence. Because the rate of kidney function decline was measured over the same time period as aTRH, it is hard to say which came first. However, the finding that declining kidney function over time is associated with the onset of aTRH increases the likelihood that kidney function is causally related to resistant hypertension. Several other arguments also favor attributing the causal role to kidney function decline. Although it is well established that poor BP control increases the risk of CKD progression, mild albuminuria or GFR reduction may precede the development of hypertension [34–37]. Moreover, insulin resistance, sodium retention, excessive activation of the renin angiotensin aldosterone system, and increased arterial stiffness [38] due to kidney function decline are pathways that might mediate the association of CKD with resistant hypertension. The high pulse pressure level in participants with aTRH provides evidence of major arterial stiffness.

This study also shows that, unlike diabetes and obesity, kidney function measured once at baseline may not predict new-onset aTRH in the elderly. This finding may argue against a causal role of kidney function in resistant hypertension. Alternatively, however, it suggests that the speed of the decline in kidney function is a better indicator of the severity of kidney impairment in the elderly than a single kidney function measurement. We have previously shown that low eGFR without specific markers of kidney damage is common in this population, particularly among women and is likely to reflect normal aging rather than true CKD [21,39]. Other studies are needed to assess whether or not low eGFR at one time point predicts new-onset aTRH in younger populations.

The large sample size of this community-based elderly population and the low rate of attrition over 4-year follow-up are major strengths of this study. Other strengths include standardized in-home BP measurements over four years and careful record of drug use, as well as standardized serum creatinine measures in a single laboratory.

This study also has limitations. First, despite its longitudinal design, it does not allow us to conclude that kidney function decline is causally related to resistant hypertension. Unidentified confounders, other than those we accounted for, may cause both kidney function decline and resistant hypertension. Second, the construction of the slope on only two points is another limitation of this study. Third, because information about adherence and drug doses was not available, pseudo-resistance could not be assessed. Similarly self-measured BP at home or ambulatory BP monitoring would have been preferable to staff-assisted BP measurements. Fourth, the absence of serum creatinine measurements in some participants at the 4-year follow-up reduced the power of the longitudinal analysis. Finally, the failure to measure albuminuria at baseline prevented us from studying its impact on new-onset aTRH. However, we were able to show that it did not confound the relation with kidney function decline, an association that is the main finding of this study.

In this elderly white population, aTRH was uncommon, but likely to have been underestimated due to treatment inertia. In addition to obesity and diabetes, this study points to rapid decline in kidney function as a major risk factor for resistant hypertension. These findings are limited to elderly, white individuals with individuals with CKD and may not apply to younger patients or to ethnic minorities. They do, however, call for increased awareness of the importance of resistant hypertension in the elderly. Identifying those who may need further investigations for secondary hypertension and enhanced treatment should be a priority to reduce adverse outcomes.

## Supporting Information

S1 File Kidney function decline and apparent treatment-resistant hypertension in the elderly. Table A: Association of kidney function estimated with MDRD, CKDEPI or BIS1 equations with the prevalence of apparent treatment-resistant hypertension at baseline. Table B: Association of kidney function decline rate with the prevalence of apparent treatment-resistant hypertension at 4-years. Table C: Association of kidney function decline rate with the prevalence of apparent treatment-resistant hypertension at 4-years in the subgroup with albuminuria. Table D: Association of kidney function decline rate with new-onset apparent treatment-resistant hypertension at 4-years. Table E: Association of kidney function decline rate (≥ 5 mL/min/1.73m²) with apparent treatment-resistant hypertension at 4-years. Table F: Association of kidney function decline rate (≥ 5 mL/min/1.73m² per year) with apparent treatment-resistant hypertension at 4-years in the subgroup with albuminuria. Table G: Association of kidney function decline rate (≥ 5 mL/min/1.73m² per year) with new-onset of apparent treatment-resistant hypertension at 4-years. Table H: Characteristics of participants according to hypertension control status at 4 years.
(TIFF)
