# Supplementary figures and images for: Kidney Function Decline and Apparent Treatment-Resistant Hypertension in the Elderly
Source: PLoS One. 2016 Jan 25;11(1):e0146056. doi: 10.1371/journal.pone.0146056 (PMC4726557; doi:10.1371/journal.pone.0146056)

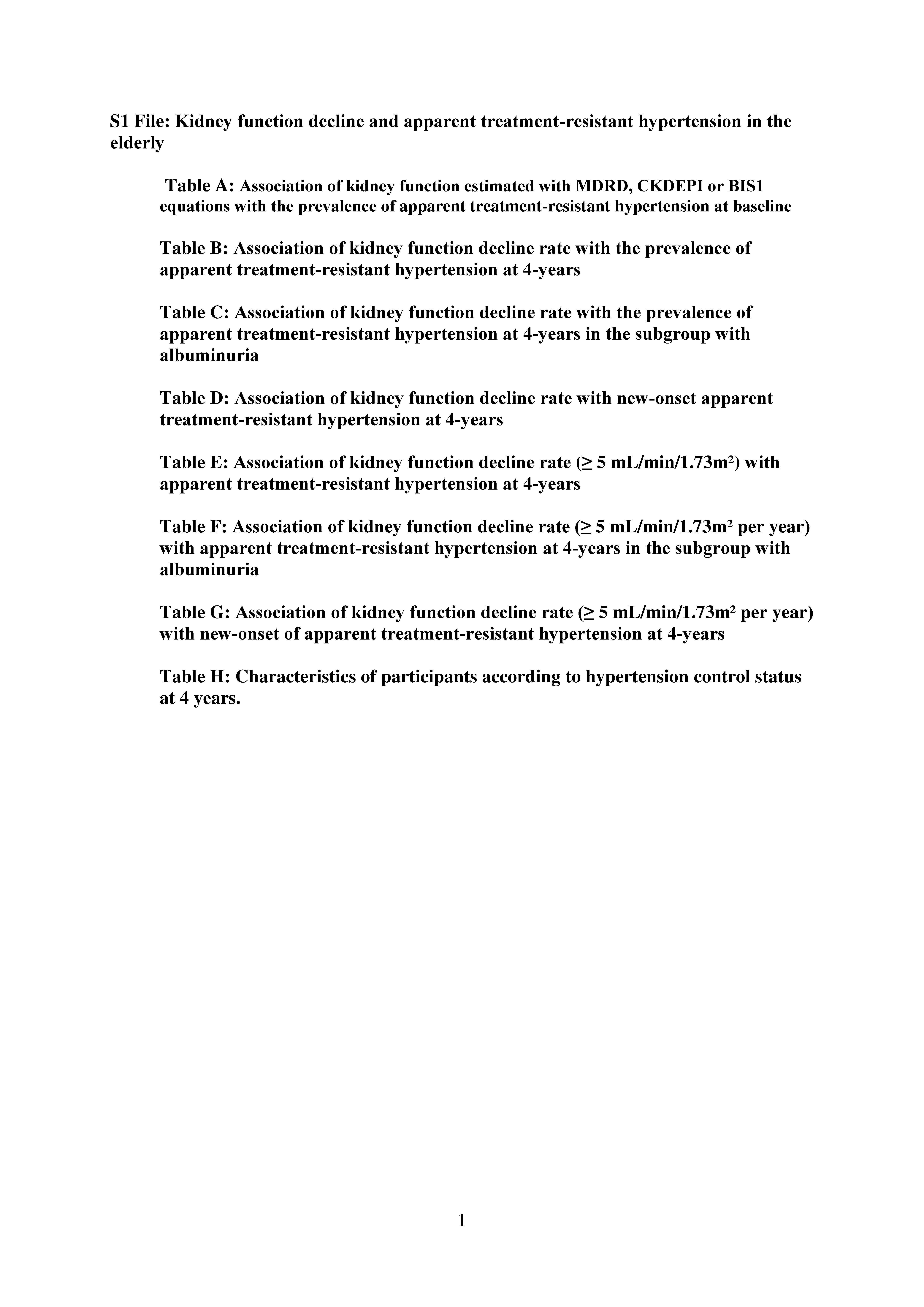

Supplement: S1 File — Table A: Association of kidney function estimated with MDRD, CKDEPI or BIS1 equations with the prevalence of apparent treatment-resistant hypertension at baseline. Table B: Association of kidney function decline rate with the prevalence of apparent treatment-resistant hypertension at 4-years. Table C: Association of kidney function decline rate with the prevalence of apparent treatment-resistant hypertension at 4-years in the subgroup with albuminuria. Table D: Association of kidney function decline rate with new-onset apparent treatment-resistant hypertension at 4-years. Table E: Association of kidney function decline rate (≥ 5 mL/min/1.73m²) with apparent treatment-resistant hypertension at 4-years. Table F: Association of kidney function decline rate (≥ 5 mL/min/1.73m² per year) with apparent treatment-resistant hypertension at 4-years in the subgroup with albuminuria. Table G: Association of kidney function decline rate (≥ 5 mL/min/1.73m² per year) with new-onset of apparent treatment-resistant hypertension at 4-years. Table H: Characteristics of participants according to hypertension control status at 4 years. (TIFF) [file pone.0146056.s001.tiff]
